# Conductivity optimisation of graphene oxide-M13 bacteriophage nanocomposites: towards graphene-based gas micronano-sensors

**DOI:** 10.1186/s11671-024-04101-w

**Published:** 2024-09-18

**Authors:** Kate Stokes, Yiwei Sun, Jarrod L. Thomas, Paolo Passaretti, Henry White, Pola Goldberg Oppenheimer

**Affiliations:** 1 School of Chemical Engineering, Advanced Nanomaterials Structures and Applications Laboratories, College of Engineering and Physical Sciences, University of Birmingham, Edgbaston, Birmingham, B15 2TT UK; 2 Paragraf Limited, Cambridge, PE28 3EB UK; 3 Institute of Cancer and Genomic Sciences, University of Birmingham, Birmingham, B15 2TT UK; 4 BAE-Systems - Air Sector, Buckingham House, FPC 267, Filton, Bristol, UK; 5 Healthcare Technologies Institute, Institute of Translational Medicine, Mindelsohn Way, Birmingham, B15 2TH UK

**Keywords:** Graphene oxide, M13 bacteriophage, Gold nanoparticles, Conductivity optimisation

## Abstract

Graphene oxide (GO) and M13 bacteriophage can self-assemble to form ultra-low density porous structures, known as GraPhage13 aerogels (GPA). Due to the insulating nature of GPA and the challenges in producing highly conductive aerogels, it is paramount to explore ways to enhance the conductivity of GPA. Herein, we have developed a method to enhance the conductivity of GPA, via the integration and optimisation of 5 nm and 20 nm diameter gold nanoparticles (AuNPs) into the aerogel structure and systematically analysed the morphology, composition and spectroscopic properties of the resulting GPA-Au nanocomposite. The fabricated GPA-Au nanocomposites exhibited remarkable increases in conductivity, with the integration of 5 nm AuNPs leading to a 53-fold increase compared to GPA, achieving a performance of up to 360 nS/cm, which is within the range suitable for miniaturised semiconductor devices. The mechanism behind the conductivity enhancement was further investigated and attributed to GO-AuNP interactions increasing the carrier density by introducing new energy levels in the GO band gap or shifting its Fermi level towards the conduction band. These findings demonstrate the potential of functionalised AuNPs to significantly improve the electrical properties of GPA, paving the way for their application in gas sensors for biological and chemical detection and a new range of advanced semiconductor devices.

## Introduction

Graphene is a monolayer of *sp*^2^-hybridised carbon atoms arranged in a honeycomb lattice, exhibiting remarkable properties including high carrier mobility, mechanical strength as well as electrical and thermal conductivity [1]. Despite these advantages, its widespread utilisation has been impeded by various challenges including the scalability issues and chemical inertness [2]. Graphene-based derivatives, such as graphene oxide (GO), offer a solution to these limitations. GO is comprised of oxygen-containing functional groups (OCFGs) including for instance, hydroxyl, carboxyl, carbonyl and epoxide, which enable the GO with the versatile capacity to interact with various compounds [3]. Notably, it can interact with biomolecules, leading to the formation of graphene-based bionano-composites, which have found applications in diverse fields such as energy storage, absorbents and drug delivery [4–7].

Recent research has demonstrated GO can interact with the M13 bacteriophage, a filamentous virus with dimensions of approximately 6.6 nm width and 880 nm length [8]. M13 is comprised of circular single-stranded DNA encapsulated by 2700 copies of the pVIII major coat protein, and the pIII, pVI, pVII and pIX minor coat proteins [9]. The interactions between GO and M13 initiate their self-assembly into a GraPhage13 hydrogel (GPH), which is subsequently dried in a vacuum to fabricate GraPhage13 aerogels (GPA), porous nanocomposites with an ultra-low density of 8.8 mg/cm^3^ and high surface area of 325 m^2^/g. The tunability of the GPA properties, either through the functionalisation of its constituents or interactions with the OCFGs in GO and chemical/genetic modification of M13, enable the routes for tailoring GPA for specific applications. With its scalable, environmentally friendly and cost-effective production, GPA is establishing itself as a versatile nanomaterial for a broad range of applications including for instance, composite scaffolds, absorbers and sensors [10, 11].

The development of highly conductive graphene-based aerogels is imperative given their immense applied potential for energy storage [12, 13], pressure [14, 15] and gas sensors [16, 17]. However, achieving high conductivity in these aerogels remains challenging. Their inherently porous nature often results in an amorphous structure with numerous defects, impeding efficient electron transfer within the material [18]. Additionally, in GPAs, the OCFGs in GO act as scattering sites, reducing the carrier mobility and leading to a low conductance of approximately 0.2 nS [19, 20]. This limitation restricts the applications of GPA in various fields, such as gas sensing, where the sensor sensitivity relies on changes in electrical conductivity caused by interactions between the sensor and the analyte [21]. Semiconducting materials within gas sensors typically exhibit conductivities ranging from 10^−7^ to 10^2^ S/cm, ensuring the detection of small conductivity changes for effective identification of gas-based biological and chemical analytes [22, 23]. However, through the functionalisation of GO and M13, it is possible to incorporate various nanomaterials into the GPA structure, offering means to modulate the conductivity. Previous research has demonstrated a 30-fold increase in its conductance though the incorporation of carbon nanotubes, highlighting the potential of this approach to produce conductive graphene-based aerogels [20].

In this study, we develop and establish a route to enhance the conductivity of GPA by integrating gold nanoparticles (AuNPs) into its micronano-structure. The interaction between GO, M13 and carboxylic acid functionalised AuNPs with diameters of 5 nm and 20 nm were investigated with ultraviolet–visible (UV–Vis) spectroscopy and the composition optimised via energy-dispersive X-ray (EDX) spectroscopy. Changes in the morphology and microstructure of the GPAs with integrated 5 nm AuNPs (GPA-Au_5nm_) and 20 nm AuNPs (GPA-Au_20nm_) were observed with scanning electron microscopy (SEM) and Raman spectroscopy, and their I-V characteristics were measured to determine if the integration of AuNPs significantly enhances conductivity. It was found that the AuNPs formed electronic interactions with GO [24, 25], enabling their integration within GPA, with the concentration of AuNPs within GPH saturating at 0.59 ± 0.01 mg/ml. The resulting GPA maintained its porous micronano-structure, and a discrete distribution of AuNPs within the optimised GPA-Au_20nm_ was evident via the SEM imaging. The insulating nature of GPA was initially confirmed with a typical conductance of 0.34 ± 0.02 nA and conductivity of 6.8 nS/cm. The integration of 20 nm and 5 nm AuNPs into GPA yielded increased conductivities of 1.9 × 10^–7^ S/cm and 3.6 × 10^–7^ S/cm, respectively, well within the range of semiconducting materials in gas sensors. SEM and Raman spectral analysis revealed that the mechanism behind the enhanced conductivity is due to the formed electronic interactions between GO and AuNPs, which introduce new energy levels in the GO band gap or shift its Fermi level towards the conduction band, thereby increasing carrier concentration.

These results further demonstrate the tunability of GraPhage13 aerogels, paving the way for the integration of additional nanomaterials to enhance their properties for specific applications. The significant improvement in GPA conductivity achieved through the incorporation of gold nanoparticles underscores the potential of these materials for advanced technologies. By enhancing conductivity while maintaining structural integrity, this study advances the understanding of nanomaterial interactions within aerogels and lays a solid foundation for the development of high-performance, multifunctional materials. The demonstrated conductivity improvements bring GPAs closer to practical use in semiconducting sensor devices, particularly for gas-based bio- and chemical sensors, with applications in diagnostics, environmental monitoring and detection of explosives [26, 27]. Furthermore, these findings emphasise the potential for further exploration of integrating additional nanomaterials into GPAs to tailor the intrinsic properties such as various types of nanoparticles to achieve even greater conductivity and other functional properties. The overall insights gained from this study provide constructive guidance, paving the way toward realising the full potential of graphene-based aerogels across a wide range of applications.

## Results and discussion

### Incorporation of gold nanoparticles

**Fig. 1:**
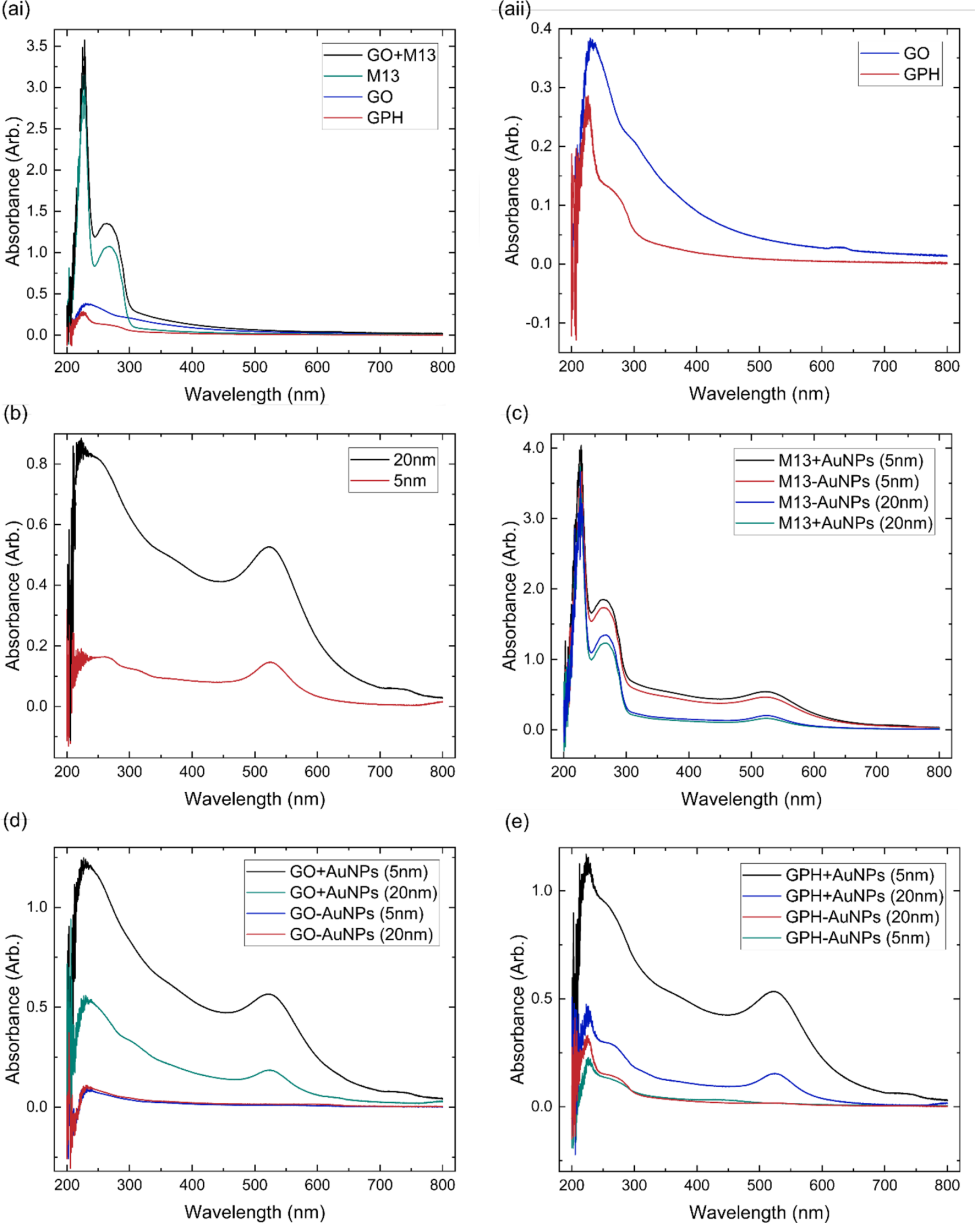
UV–Vis spectra of the supernatants generated from mixtures of graphene oxide (GO), M13 bacteriophage and carboxylic acid functionalised gold nanoparticles (AuNPs) with diameters of 5 nm and 20 nm. The spectrum of GO, M13 and GPH supernatants in comparison to the addition of the GO and M13 spectra, are shown in **(ai)** with **(aii)** zoom-in on the features of the GO and GPH spectra. **b** The supernatants produced by centrifuging 5 nm and 20 nm AuNPs. **c** Comparison of the addition of M13 and Au supernatants (M13 + AuNPs) after mixing M13 and Au (M13-AuNPs) with 5 nm and 20 nm AuNPs. **d** The supernatants from the addition and mixtures of graphene oxide (GO) with 5 nm and 20 nm AuNPs and **e** the UV–Vis spectrum of mixing GraPhage13 hydrogel (GPH) and 20 nm and 5 nm AuNPs

The interactions between AuNPs and GPAs were investigated via the UV–Vis spectroscopy. In the initial stages of GPA production, GO and M13 are introduced into a buffer, enabling their self-assembly. Following this, centrifugation separates and isolates the components from solution. Through removing 90% of the supernatant and re-suspending the pellet, the GraPhage13 hydrogel (GPH) is generated. However, the supernatant itself provides valuable insights. By combining GO and AuNPs (GO-AuNPs), M13 and AuNPs (M13-AuNPs) and all the components (GPH-AuNPs), followed by centrifugation, supernatant analysis and comparison with the UV–Vis spectra of the individual components within the solution, we determine whether the components interacted or eliminated from the solution or whether they remain unreactive and remain present in solution (Fig. 1).

The UV–Vis spectra (Fig. 1a) illustrate the characteristics of the supernatants produced after centrifugation of GO, M13 and GPH, compared to the combined spectra of GO and M13 (GO + M13). In the GO supernatant spectrum, a peak at 230 nm indicates the π → π* transition of C=C bonds, while a shoulder at 310 nm suggests n → π* transitions of C=O bonds [28]. The M13 spectrum exhibits a prominent absorption band between 200–230 nm, attributed to the π → π* transitions of peptide bonds, with a distinct peak at 269 nm resulting from the major coat protein pVIII and viral DNA [10]. The GPH supernatant spectra resembles the spectrum for GO with a more pronounced peak at 269 nm due to the presence of the M13. When comparing GPH to GO + M13, the absorption notably decreases, *e.g.,* the peak at 269 nm decreases from 1.1 to 0.12. This indicates that the interactions between GO and M13, namely the pH-dependent electrostatic interactions between the negatively charged carboxylic acids on GO and the positively charged groups of the N-terminus and K_8_ residues of the M13, produce GO-M13 pellet, which precipitates from solution to produce GPH [8, 10].

The UV–Vis spectra of 5 nm and 20 nm AuNPs supernatants (Fig. 1b) display characteristic peaks around 524 nm and 522 nm, respectively, corresponding to localised surface plasmon resonance. While both AuNPs exhibit similar absorption behaviour, their interaction with M13 is negligible, as indicated by consistent M13 peak absorbance in the presence and absence of AuNPs (Fig. 1c). Conversely, the combination of GO and AuNPs leads to a significant reduction in AuNP absorption (Fig. 1d), indicating their interaction and subsequent precipitation [29]. Similarly, AuNPs integrate into GPH, resulting in decreased absorption peaks for both AuNPs and GPH components compared to their individual spectra. This reduction in absorbance upon mixing with GPH signifies the interactions between AuNPs and GO, facilitating their precipitation from the solution. This hybridisation, involving the carboxyl groups on the AuNPs and the oxygen-containing functional groups on GO [24, 25], creates a new composite material where the electronic and structural properties of GO are significantly modified by the presence of AuNPs.

### Optimisation of GPA-Au

**Fig. 2:**
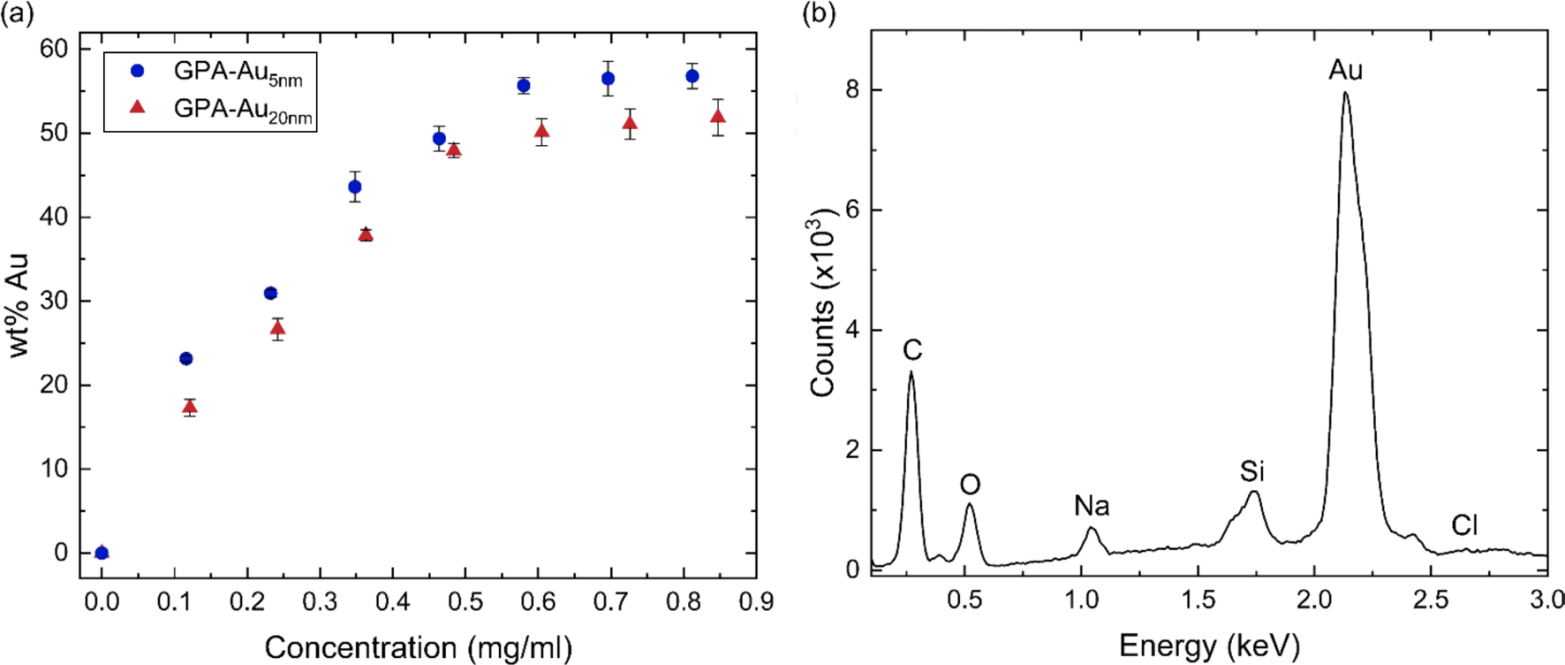
**a** The relationship between the concentration of gold nanoparticle (AuNPs) solution utilised to produce the GraPhage13 hydrogel (GPH), subsequently dried to form the GraPhage13 aerogel (GPA) and the resulting weight percentage of Au in the aerogel containing AuNPs with diameters of 5 nm (GPA-Au_5nm_) and 20 nm (GPA-Au_20nm_). **b** Representative EDX spectrum of GPA-Au

To determine the optimal concentration of the 5 nm and 20 nm AuNPs for integration into GPA, GPH-Au with varying concentrations of AuNPs were synthesised and the resulting GPA-Au analysed using energy dispersive x-ray spectroscopy (EDX). The weight percentage of gold incorporated into GPA was studied across different AuNP concentrations (Fig. 2a).

**Fig. 3:**
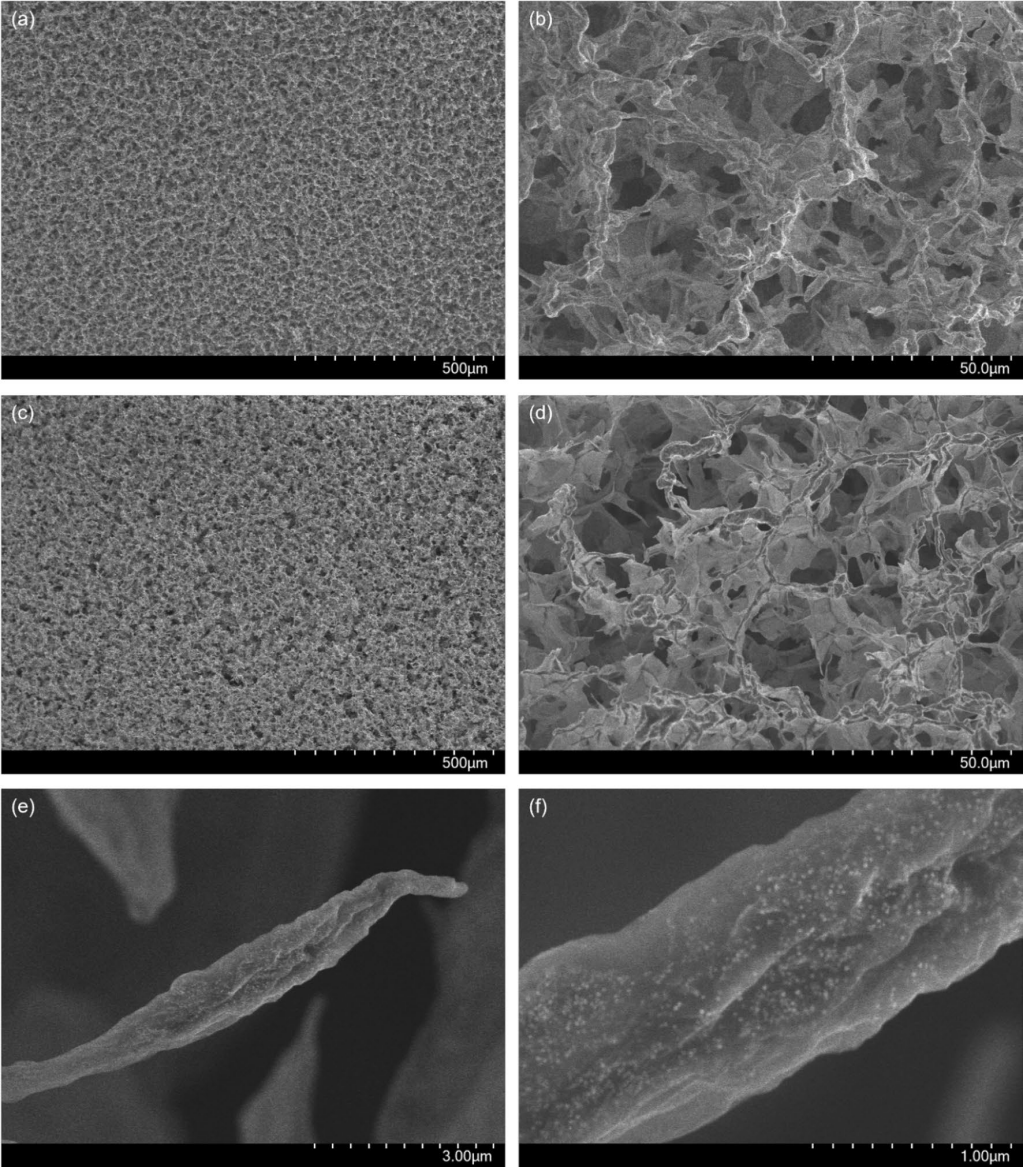
SEM images of GraPhage13 aerogels functionalised with **a–b** 5 nm AuNPs and **c–f** 20 nm AuNPs

As the concentration of AuNPs increases, the wt% Au also increases until reaching a plateau, indicating saturation of GPH and no further increase in AuNP concentration within GPA. For GPA-Au_20nm_, saturation occurs at approximately 0.61 mg/ml, resulting in 50 ± 2 wt% Au, while for GPA-Au_5nm_, saturation occurs at approximately 0.58 mg/ml, yielding 56 ± 1 wt% Au. The higher maximum wt% Au for GPA-Au_5nm_ compared to GPA-Au_20nm_ may be attributed to the larger size of 20 nm AuNPs blocking some of the binding sites on GO, resulting in lower Au content. Additionally, the smaller 5 nm AuNPs may integrate more effectively within the micro-nanostructure. A representative spectrum of optimised GPA-Au is shown in Fig. 2b, with SEM images of resulting GPA-Au in Fig. 3, where the presence of 20 nm AuNPs is clearly visible **(**Fig. 3e–f**).**

### In-depth Raman spectroscopy

From our previous Raman analyses of GPA [20, 30], at least two distinctive peaks were expected, the D-mode, which has contributions from both defective *sp*^2^ carbon, and *sp*^3^ amorphous or disordered carbon, and the G-mode, stemming from the *sp*^2^ bond stretching. Other peaks which may be present for GPA are the G^−^ and D^’^ modes. The G^−^ mode typically appears a few tens of wavenumbers lower than the G mode and is associated with significantly modified *sp*^2^ bonds. In GPA, this mode could arise from consistently softened *sp*^2^ bonds, such as those resulting from intensive functionalisation in GO, or from the bent graphene planes after its assembly with M13. Additionally, it may result from charge transfer between GO and M13, leading to a reduction in the energy of a portion of transverse optical (TO) or longitudinal optical (LO) phonons. The D’ mode arises from crystal defects due to *sp*^2^ bond functionalisation in GO [20, 30].

**Fig. 4:**
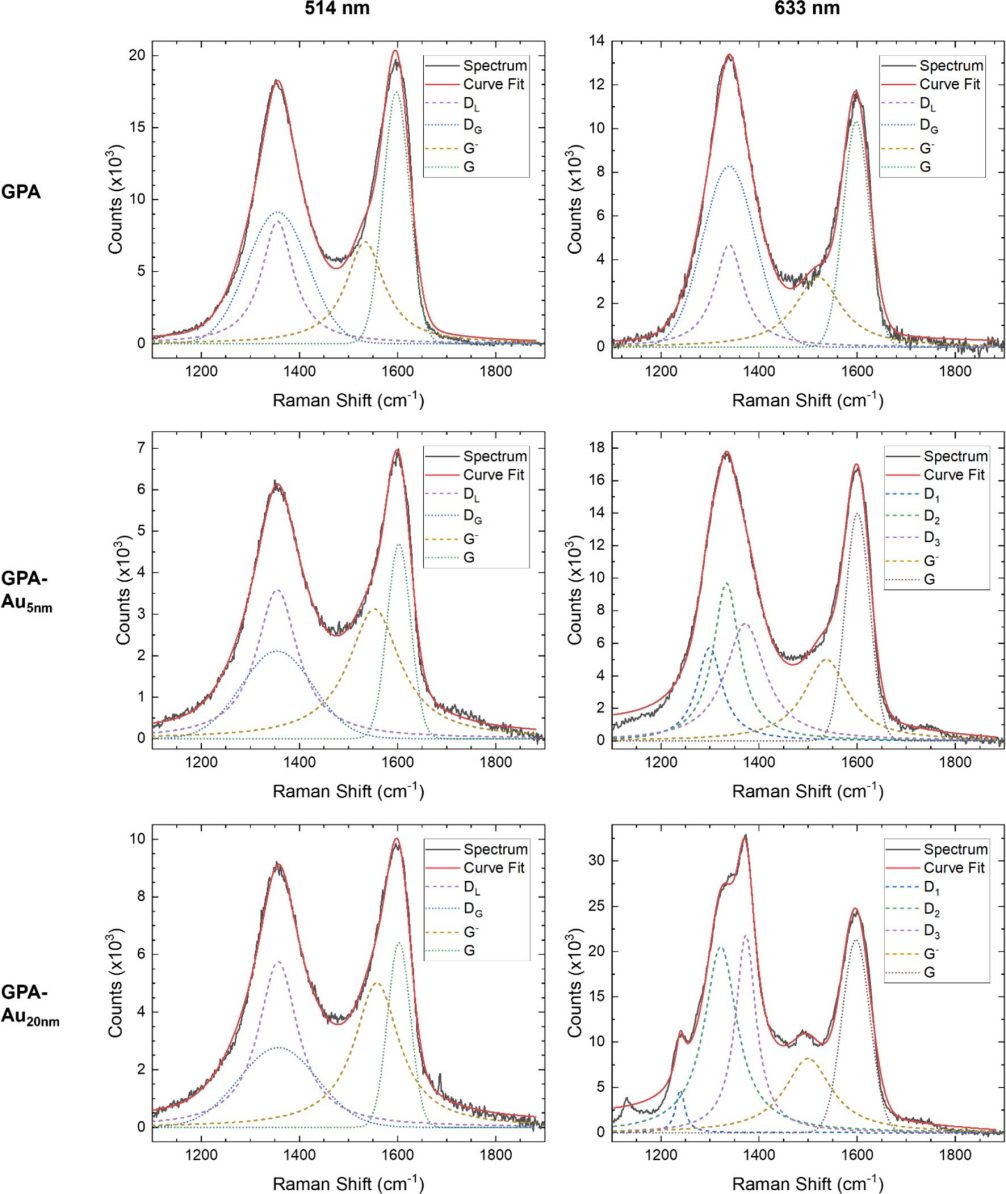
Raman spectra of GPA, GPA-Au_5nm_ and GPA-Au_20nm_ at wavelengths of 514 nm and 633 nm, with the overall curve fit. The Lorentzian and Gaussian fits are given by dashed and dotted lines, respectively

Herein, Raman spectroscopy was conducted on GPA, GPA-Au_5nm_ and GPA-Au_20nm_ using excitation wavelengths of 514 nm and 633 nm. The purpose of using two lasers was to identify whether the observed Raman features in the D mode range originated from the D mode associated with C–C *sp*^*2*^ bonds or the *sp*^*3*^ Raman mode, as the D mode frequency shifts with laser excitation, whereas a *sp*^*3*^ Raman peak is non-dispersive peak [31–33]. The Bayesian information criterion (BIC) was utilised to determine the optimal objective models for fitting each spectrum (Fig. 4) [20].

Under 514 nm excitation, the D features of all three samples consist of a single peak at 1355 cm^−1^ (Table 1), despite changes in peak shape and frequency shifts. This peak is approximately 20 cm^−1^ higher than the *sp*^*3*^ peak, with its expected Raman shift of 1332 cm^−1^, hence attributed to the D mode. The optimal peak fit for the D-mode was generated by fitting one Lorentzian and one Gaussian peak, termed D_L_ and D_G_ respectively, at the same Raman shift of 1355 cm^−1^, generating a mixed Gaussian–Lorentzian line-shape [34, 35].

**Table 1:** Raman shift, full width half maximum (FWHM) and intensity of the Raman peaks present in spectra of GPA, GPA-Au_5nm_ and GPA-Au_20nm_, obtained at a wavelength of 514 nm

Peak	GPA			GPA-Au_5nm_			GPA-Au_20nm_		
	Raman Shift (cm^−1^)	FWHM (cm^−1^)	Intensity (cm^−1^)	Raman Shift (cm^−1^)	FWHM (cm^−1^)	Intensity (cm^−1^)	Raman Shift (cm^−1^)	FWHM (cm^−1^)	Intensity (cm^−1^)
D_L_	1356.5 ± 0.4	39 ± 2	(1.1 ± 0.1) × 10^6^	1354.6 ± 0.3	50 ± 2	(7.0 ± 0.4) × 10^5^	1355.7 ± 0.3	48 ± 2	(9.3 ± 0.6) × 10^5^
D_G_		62 ± 1	(9.6 ± 0.6) × 10^3^		74 ± 1	(2.9 ± 0.2) × 10^3^		73 ± 2	(3.3 ± 0.2) × 10^3^
G^−^	1539 ± 3	44 ± 3	(1.1 ± 0.1) × 10^6^	1553 ± 4	62 ± 3	(8.2 ± 0.8) × 10^5^	1550 ± 2	53 ± 1	(8.2 ± 0.2) × 10^5^
G	1598.3 ± 0.6	26.3 ± 0.5	(1.84 ± 0.03) × 10^3^	1602.4 ± 0.5	24.1 ± 0.5	(6.3 ± 0.2) × 10^3^	1601.6 ± 0.4	24.5 ± 0.4	(8.2 ± 0.1) × 10^3^

Under 633 nm excitation, GPA also exhibits a single D peak similar to that observed under 514 nm, however its position downshifts to 1338.7 cm^−1^. The D mode is activated by a defect-induced double resonance process, involving the energy of the LO phonon at the K point in the Brillouin zone. The presence of a Kohn anomaly at the K point causes the highly dispersive nature of the D peak [36].

The splitting of the D profile into three peaks, D_1-3_, is clearly visible for GPA-Au_20nm_ under 633 nm and becomes evident for GPA-Au_5nm_ through comparing BIC values. This split appears following the introduction of AuNPs and is more pronounced with 20 nm AuNPs. Meanwhile, this split does not appear under 514 nm. These observations indicate that the split peaks are likely modified D peaks, becoming visible due to being in resonance under 633 nm. The integration of AuNPs into GPA modifies the *sp*^*2*^-network, altering the phonon dispersion curve, particularly the LO branches. This results in a significant shift in the G^−^ mode (LO at the Gamma point) (Tables 1–2) and an obvious splitting of the D mode (LO at the K point), as captured by the 633 nm laser. The modification of the dispersion curve, indicative of perturbations to the *sp*^*2*^ network of GPA, is more substantial with 20 nm AuNPs than with 5 nm AuNPs [37, 38].

**Table 2:** Raman shift, full width half maximum (FWHM) and intensity of the Raman peaks for GPA, GPA-Au_5nm_ and GPA-Au_20nm_, obtained at a wavelength of 633 nm

Peak	GPA			Peak	GPA-Au_5nm_			GPA-Au_20nm_		
	Raman Shift (cm^−1^)	FWHM (cm^−1^)	Intensity (cm^−1^)		Raman Shift (cm^−1^)	FWHM (cm^−1^)	Intensity (cm^−1^)	Raman Shift (cm^−1^)	FWHM (cm^−1^)	Intensity (cm^−1^)
D_L_	1338.7 ± 0.5	38 ± 2	(7 ± 1) × 10^5^	**D**_**1**_	1229 ± 4	32 ± 4	(6.4 ± 0.4) × 10^5^	1239.5 ± 0.8	11 ± 1	(1.9 ± 0.3) × 10^5^
D_G_		53.7 ± 0.8	(1.1 ± 0.1) × 10^4^	**D**_**2**_	1335 ± 2	33 ± 8	(1.1 ± 0.6) × 10^6^	1321 ± 1	40 ± 1	(3.0 ± 0.2) × 10^6^
_-_	-	-	-	**D**_**3**_	1374 ± 8	50 ± 4	(1.1 ± 0.4) × 10^5^	1372.5 ± 0.5	26.6 ± 0.9	(2.1 ± 0.1) × 10^6^
G^−^	1522 ± 3	64 ± 4	(9.6 ± 0.8) × 10^6^	**G**^**−**^	1537 ± 4	53 ± 5	(9 ± 1) × 10^5^	1501 ± 2	58 ± 3	(1.8 ± 0.1) × 10^6^
G	1598.3 ± 0.3	27.0 ± 0.4	(1.4 ± 0.2) × 10^4^	**G**	1600.5 ± 0.5	25.1 ± 0.4	(1.52 ± 0.03) × 10^4^	1597.3 ± 0.3	28.6 ± 0.4	(2.44 ± 0.03) × 10^4^

Additionally, the 633 nm Raman spectrum of GPA-Au_20nm_ shows the highest peak intensities, with a maximum intensity of (3.7 ± 0.2) × 10^4^ counts, in comparison to (1.9 ± 0.1) × 10^4^ counts for GPA-Au_5nm_. This might be attributed to the localised surface plasmon resonance (LSPR) exhibited by AuNPs. When electromagnetic radiation interacts with the conduction electrons on the surface of the AuNPs, it causes the electrons to oscillate at the same frequency as the incident light. At the resonant frequency, this results in an amplified local electromagnetic field, leading to an increased Raman signal [39]. For AuNPs with diameters smaller than 50 nm, collisions between the electrons and the particle surface decreases their mean free path. This dampens the LSPR proportionally to the particle diameter, thereby reducing the Raman intensity [40].

To gain further insight into Raman spectra, the intensity ratio of D to G peaks (*I*_*D*_*/I*_*G*_) was calculated (Table 3), to give a measure of the concentration of defects and disorder within a sample [41].

**Table 3:** The intensity ratio of D-peak to G-peak (*I*_*D*_*/I*_*G*_) of Raman peaks for GPA, GPA-Au_5nm_ and GPA-Au_20nm_, at wavelengths of 514 nm and 633 nm

Parameter	GPA		GPA-Au_5nm_		GPA-Au_20nm_	
	514 nm	633 nm	514 nm	633 nm	514 nm	633 nm
*I*_*D*_*/I*_*G*_	0.91 ± 0.08	0.81 ± 0.08	0.86 ± 0.07	3.1 ± 0.6	1.15 ± 0.05	2.9 ± 0.1

At the 633 nm laser excitation, the integration of AuNPs into GPA increases *I*_*D*_*/I*_*G*_ due to an increase in the intensity of the D-profile, as many modified D modes become resonant. This D-profile splitting indicates that the AuNPs introduce defects into the structure, likely resulting from interactions between GPA and the AuNPs [42, 43]. Furthermore, *I*_*D*_*/I*_*G*_ appears to be independent of the diameter of the AuNPs. This may be attributable to the GPA-Au composites being produced using the same fabrication process, ensuring similar chemical and structural properties. Additionally, the defects are intrinsic to the GPA structure and the interactions between GO and the carboxylic groups on the AuNPs, making them independent of the AuNP diameter. Moreover, the increase in *I*_*D*_*/I*_*G*_ through the integration of AuNPs suggests an increase in the electrical conductivity [44]. These trends are not observed at the 514 nm laser excitation. There is little change in *I*_*D*_*/I*_*G*_ between GPA and GPA-Au_5nm_, and only a slight increase for GPA-Au_20nm_. The 514 nm laser is not able to induce resonance in the modified D modes and therefore, structural changes to GPA due to the presence of AuNPs are less detectable [37].

### Conductivity of GPA and GPA-Au

**Fig. 5:**
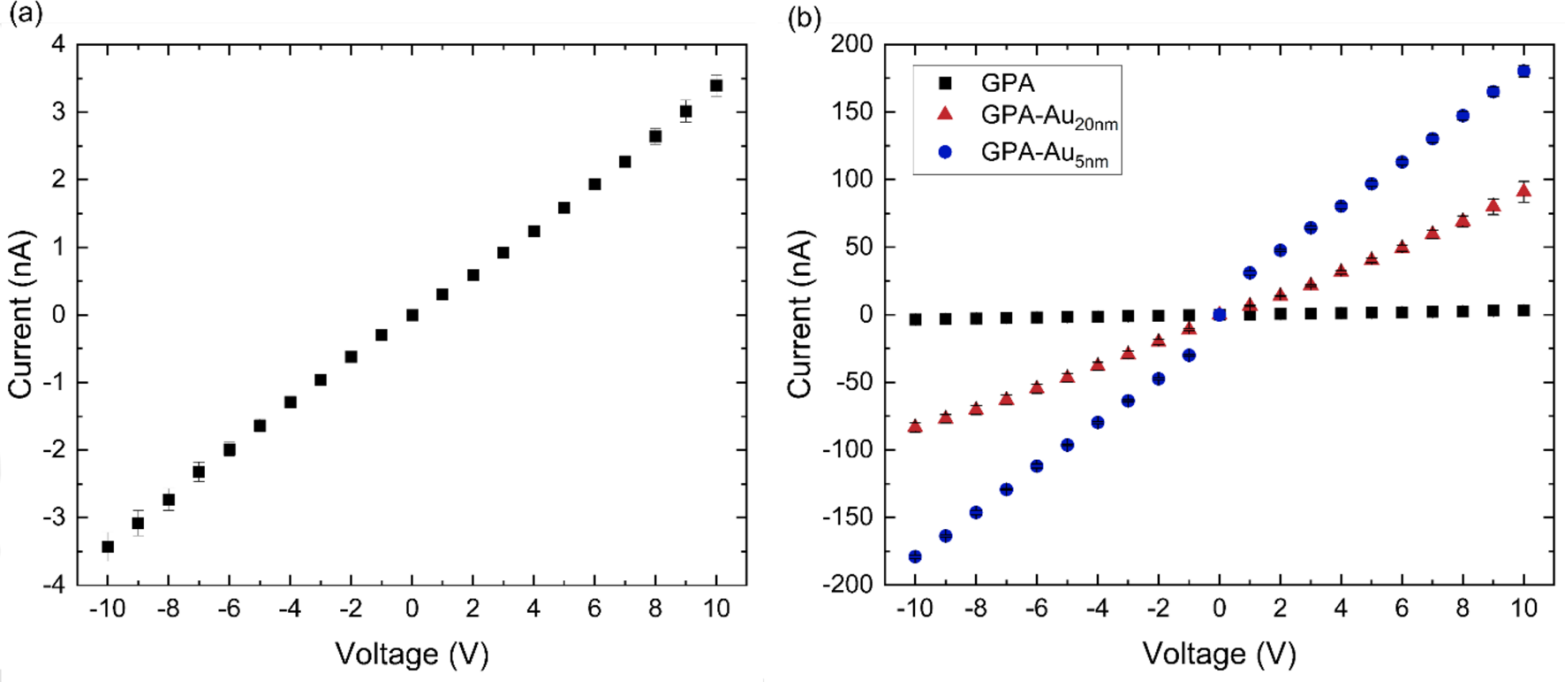
**a** I–V characteristic of GPA and **b** comparison of the I–V characteristics of GPA, GPA-Au_20nm_ and GPA-Au_5nm_

The I-V characteristic of GPA (Fig. 5a) aligns closely with previous findings [20] and confirms the insulating nature of GPA, with a maximum current of 3.4 ± 0.2 nA at 10 V. This leads to a conductance of 0.34 nS and a conductivity of 6.8 nS/cm, two orders of magnitude below the minimum conductivity for semiconducting materials utilised within gas sensors. GPA-Au_20nm_ (Fig. 5b) exhibits a significantly enhanced conductivity, reaching 90 ± 8 nA at 10 V, marking a 26-fold increase over GPA and generating a conductivity of 190 nS/cm, achieving a value within the range for semiconducting materials in gas sensors. The conductivity was enhanced further through the integration of 5 nm AuNPs, with GPA-Au_5nm_ demonstrating a current of 180 ± 4 nA at a bias of 10 V, twice that of GPA-Au_20nm_ and representing a 53-fold increase compared to GPA. This is equivalent to a conductance of 18 nS and a conductivity of 360 nS/cm, representing a significant step towards the integration of GPA in sensing devices.

Through the morphological studies via the SEM and microstructural changes via Raman spectroscopy, the mechanism by which the conductivity of GPA is enhanced through the integration of AuNPs has been subsequently determined. Since SEM images demonstrate a discrete distribution of AuNPs across the GPA, the current must be transmitted through GPA itself, rather than exclusively through the AuNPs. Additionally, since concentration of 5 nm and 20 nm AuNPs introduced into GPH was equal, and yet the GPA-Au_5nm_ proved to be twice as conductive as GPA-Au_20nm_, the presence of Au cannot be the sole cause of the increased conductivity. Therefore, the conductivity enhancement must be through the interactions between GO-M13 and the AuNPs. The greater increase in conductivity observed for the integration of 5 nm AuNPs compared to the 20 nm AuNPs is likely due to the 5 nm AuNPs possessing a higher surface-to-volume ratio, facilitating a higher surface area for interactions between GO and the AuNPs [45].

There are two mechanisms by which the AuNPs interacting with GO-M13 could enhance the conductivity of the resulting GPA, either by increasing the carrier density or by increasing the carrier mobility [46]. The presence of scattering at functional sites within GO significantly reduces its carrier mobility. Reducing GO to reduced graphene oxide eliminates some of the functional groups and aids in the dispersion of the free carriers, increasing their mobility [19].

This would be evident in Raman spectra via a redshift in the G-mode position as the concentration of *sp*^*2*^ bonds increases and a reduction in *I*_*D*_/*I*_*G*_ resulting from a reduced level of defects [47]. Similarly, the bonding of the AuNPs to GO would reduce the concentration of available functional groups and therefore the G-mode position and *I*_*D*_/*I*_*G*_ would be expected to decrease. Conversely, a significant increase in *I*_*D*_/*I*_*G*_ is observed and the G-mode remains relatively constant when comparing GPA to GPA-Au_5nm_ and GPA-Au_20nm_. Therefore, an increase in the carrier density is most probable mechanism behind the conductivity enhancement. An increase in the carrier density through the introduction of AuNPs into GPA likely originates from alterations to the band gap of GO, which has a relatively wide band gap of 2.2 eV, rendering GO an insulator [48, 49]. The presence of AuNPs introduces new energy states within this band gap, shift the Fermi level towards the conduction band, or a combination of these effects, resulting in an increased conductivity [50–53].

Smaller AuNPs possess a higher surface-to-volume ratio, which increases the available surface area for interaction with GO, leading to a more stable dispersion of the AuNPs within the solution. This stable dispersion promotes a higher density of GO-Au interactions, which is essential for enhancing the carrier density through the previously discussed mechanism, thereby improving the conductivity. Consequently, GPA-Au_5nm_ exhibits a higher conductivity compared to GPA-Au_20nm_ [54, 55].

Given the mechanism by which AuNPs enhance the conductivity of GPA, a positive correlation between the amount of AuNPs incorporated into GPA and the resulting conductivity is expected. The conductivity improvement depicted in Fig. 5 corresponds to the maximum saturation of Au wt% in GPA. This implies that increasing the AuNP loading beyond this saturation point will not result in further conductivity gains. Conversely, reducing the AuNP load decreases the Au wt% in GPA, thereby diminishing the conductivity enhancement. If the AuNP load is sufficiently reduced, the GPA will eventually return to its insulating state in the absence of AuNPs.

## Conclusions

A novel method has been developed and validated for transforming insulating graphene oxide-based aerogels into conductive materials suitable for semiconductor sensors. This advancement paves the way for future integration into electronic systems and the development of advanced graphene-based sensors. The integration of 5 nm and 20 nm diameter carboxylic acid-functionalised gold nanoparticles (AuNPs) into GraPhage13 aerogels (GPA) was systematically investigated using UV–Vis spectroscopy and EDX. UV–Vis spectra revealed interactions between graphene oxide (GO) and AuNPs, facilitating their integration into the GraPhage13 hydrogel (GPH), the precursor for GPA. EDX measurements determined the saturation concentration of AuNPs within GPH. The optimal GPA-Au with the maximum concentration of AuNPs was further analysed using SEM, Raman spectroscopy, and conductivity measurements. SEM analysis confirmed the integration of AuNPs into the GPA micro-nanostructure, with Raman spectra showing distinct changes in the D-mode and G-mode due to the presence of AuNPs. These changes were more pronounced in GPA-Au_20nm_ compared to GPA-Au_5nm_, indicating that AuNPs modify the *sp*^*2*^ carbon network and phonon dispersion in GO. Conductivity measurements revealed a significant increase in GPA conductivity, transitioning from an insulator with a conductivity of 6.8 nS/cm to 190 nS/cm and 360 nS/cm for GPA-Au_20nm_ and GPA-Au_5nm_, respectively. This study highlights the potential to tune GPA properties through nanoparticle integration, offering possibilities for further customisation with various nanomaterials and modifications of M13 viral building blocks. The significant enhancement of GPA conductivity through AuNP integration makes it a promising candidate for integration into miniaturised devices for applications such as energy storage, gas, and pressure sensors.

## Materials and methods

### Propagation and purification of M13 bacteriophage

The method is described by Stokes *et* al. [8]. Briefly, One Shot TOP10F’ Chemically Competent *Escherichia coli* (*E. coli*) (Thermo Fisher Scientific) cells were inoculated on nutrient agar plates and subsequently, incubated overnight at 37 °C. The cultivated cells were depositing into 50 ml falcon tubes with nutrient broth (NB) and tetracycline in ethanol (Sigma) (TCN), to a final concentration of 5 μg/ml. The tubes were incubated overnight in a shaker incubator at 37 °C, 150 rpm and then added to further NB and TCN in conical flasks. M13 (0.05 mg) from a stock solution was incubated overnight in shaker incubator. The solution was centrifuged twice (Beckman Coulter, JLA 10.5) to remove the *E. coli* cells, mixed with a 25% polyethylene glycol (PEG) 6000 and 2.5 M NaCl (PEG-NaCl) and stirred on ice for 90 min. The solution was centrifuged to produce a while pellet, which was resuspended in DIW and centrifuged in a microcentrifuge (SciSpin MICRO). The addition of PEG-NaCl and leaving the solution on ice for 60 min enabled the M13 to precipitate from solution and centrifuging yielded the M13 pellet, resuspended in DIW.

### Fabrication of GraPhage13 aerogels

The methodology is described by Passaretti *et* al. [10]. M13 and GO (Graphene Supermarket, SKU-HCGO-W-175ML) were introduced into a 10 mM citrate buffer with a pH of 4.9, both reaching a concentration of 0.3 mg/ml. Subsequently, the solution was thoroughly mixed using an orbital shaker at 150 rpm for 15 min, followed by centrifugation at 15,000 rpm for 1 min. This step yielded the formation of a GO and M13 pellet. By eliminating 90% of the supernatant and re-suspending the pellet, GraPhage13 hydrogel (GPH) materialises. The next phase included depositing 100µL of GPH onto a cleaned glass substrate, subsequently subjected to vacuum drying for 60 min, culminating in the creation of the GPA product. For the fabrication of GPA-Au, 250 μL of 5 nm (765430-1ML, core diameter 3-7 nm) or 20 nm (765511-1ML, core diameter 18–22 nm) carboxylic acid functionalised gold nanoparticles (Sigma-Aldrich) were introduced into the citrate buffer with GO and M13.

### UV–Vis spectroscopy

An Aligent Cary 60 UV–Vis spectrophotometer was used to determine the concentration of M13 [8]. Briefly, a baseline spectrum was taken in quartz cuvette with 1 cm path length and the M13 in DIW was analysed. The characteristic spectrum of M13 presented a local minimum in absorbance at 245 nm, a local maximum at 269 nm and a baseline at 350 nm. The concentration of M13 was calculated with the Beer-Lambert Law and an extinction coefficient of 3.84cm^2^/mg at 269 nm [56]. UV–Vis was also employed to analyse the supernatants of GO, M13 and AuNPs to determine whether the components had interacted and precipitated from solution or remained in the supernatant. To replicate the conditions required to produce GraPhage13 aerogels, the supernatants for GO and/or M13 were generated (Sect. 4.2) but instead the supernatant was deposited into quartz cuvette and spectra recorded. For producing solutions of AuNPs, 10ul of citrate buffer was removed and 10ul of AuNPs added.

### Scanning *electron* microscopy and energy dispersive X-ray spectroscopy

The morphology of GPA and GPA-Au were compared with a Hitachi SU5000 scanning electron microscope at 1-5 kV to lower charging effects. The composition of these aerogels was determined with energy-dispersive X-ray spectroscopy with a Hitachi TM3030 microscope possessing Oxford Instruments Swift ID.

### Raman spectroscopy

Raman Spectrometer (Renishaw *inVia* Qontor) was calibrated with Silicon, characterised by a Raman peak at 520 cm^−1^ [57]. GPA, GPA-Au_5nm_ or GPA-Au_20nm_ were then placed into the chamber and Raman spectra were obtained at both 514 nm and 633 nm wavelengths. The 633 nm spectra were recorded with × 50 objective, 5% laser power (719 µW), 10 s acquisition time and 10 accumulations and the 514 nm spectra were recorded with a × 20 objective, 5% laser power (1.03 mW), 60 s acquisition time and 3 accumulations. The spectra were processed with Python, subtracting the baseline and optimising the peak fitting.

### Conductivity measurements

The conductivity of the GPAs was measured with a Keithley 617 Electrometer. 100 µL of GPH were dried on 10 × 10 mm glass substrates with conductive metallic tape (RS Pro) spanning 25 mm at each end, allowing the current to pass through 5 mm of the resulting GPA. An electrical connection between the GPA and conductive tape was ensured with silver conductive lacquer (RS Pro). To minimise current leakage, triaxial cables were used and the set-up was placed within a grounded outer electromagnetic interference shielding enclosure.

## Data Availability

Data is provided within the manuscript. Raw data of this study is available from the corresponding author upon request.
